# Initial Clinical Outcomes in the Treatment of Brain Metastasis With Gyroscopic Radiosurgery at a Single Institution

**DOI:** 10.7759/cureus.97282

**Published:** 2025-11-19

**Authors:** Tamra-Lee McCleary, Neelan J Marianayagam, Lorenzo Yuan, Kelly H Yoo, Vashisht Sekar, Amit Persad, David J Park, Antonio Meola, Peter D Klassen, Steven D. Chang

**Affiliations:** 1 Department of Neurosurgery, Stanford University School of Medicine, Stanford, USA; 2 Department of Neurosurgery, University of Florida College of Medicine – Jacksonville, Jacksonville, USA; 3 Department of Neurosurgery, Bonifatius Hospital, Lingen, DEU

**Keywords:** brain metastasis, gyroscopic radiosurgery, patient outcomes, stereotactic radiosurgery, zap-x

## Abstract

Introduction: Brain metastases occur in approximately 20% to 40% of individuals annually diagnosed with cancer. This poor prognostic feature can severely reduce life expectancy and quality of life. Stereotactic radiosurgery (SRS) has been shown to provide excellent tumor control and clinical outcomes. ZAP-X (Zap Surgical Systems Inc., San Carlos, CA, USA) is an innovative self-shielded gyroscopic stereotactic radiosurgery (GRS) system to help further address these needs. In this study, we evaluate the clinical outcomes of patients with brain metastases who have undergone treatment with ZAP-X in a single institution.

Methods: Medical records of all patients with brain metastases treated with GRS between May 2021 and May 2023 were reviewed as a retrospective case series. Demographics, clinical symptoms, treatment trajectory, and follow-up data were collected and analyzed.

Results: A total of 46 patients with 140 combined brain metastases were included in this cohort. Of them, 58.7% (N=27) were female patients, and the mean age was 63 years (range 36-82). The primary pathologies were non-small cell lung cancer (NSCLC; N22, 47.8%), breast cancer (N11, 23.9%), melanoma (N5, 10.9%), and others (N8, 17.4%). The prescription dose was 18 Gy if the patient previously received whole-brain radiotherapy (WBRT); otherwise, 20 Gy. All patients received dexamethasone (3x4 mg PO). The most common symptoms included headache (45.7%), motor disturbances (30.4%), and seizures (21.7%). Mean follow-up time was 8.5 months (range 0-19). Systemic response using Response Evaluation Criteria in Solid Tumors (RECIST) criteria was 17.3% complete response, 10.9% partial response, 45.7% stable disease, and 26.1% progression of disease. Tumor control was 73.9% according to the Response Assessment in Neuro-Oncology (RANO) criteria for brain metastases.

Conclusions: GRS is a novel image-guided linear accelerator-based, self-shielding gyroscopic radiosurgery platform that does not require a vault. This study shows that GRS achieves comparable results to traditional SRS platforms in the treatment of brain metastases.

## Introduction

Stereotactic radiosurgery (SRS) is a well-established platform for the accurate delivery of highly focused radiation. with minimal exposure to surrounding structures. SRS has replaced whole-brain radiotherapy (WBRT) as the standard of care in the treatment of brain metastasis [1]. In general, SRS shows reduced toxicity and side effect profile when compared to WBRT. Studies have shown that there is no benefit to overall survival in patients treated with WBRT combined with SRS compared to SRS alone [2]. In addition, patients have better post-treatment cognitive outcomes with SRS when compared to WBRT [3].

The important therapeutic role of SRS has promoted its evolution with improved capabilities for diverse applications [4]. One of the major drawbacks of both Gamma Knife (Elekta AB, Stockholm, Sweden) and most linear-accelerator (LINAC)-based SRS systems is the need for them to be installed in radiation-safe rooms or vaults. This requirement is costly and even prohibitive for some institutions. ZAP-X (ZAP Surgical Systems, Inc., San Carlos, CA, USA) addresses the growing need for accessible radiosurgical therapies by developing a gyroscopic radiosurgery device (GRS) that does not require a radiation-safe vault. The aim of this study is to evaluate the outcomes of patients with brain metastasis who have undergone GRS treatment. This study presents the largest cohort, to date, of patients with brain metastasis treated with GRS.

An abstract of this article was previously presented as a poster at the 2024 Congress of Neurological Surgeons Annual Meeting in Houston, TX, USA, in October 2024.

## Materials and methods

Institutional Review Board approval (Stanford University IRB-61 issued approval 67410) was obtained prior to the retrospective review of medical records. All records of patients treated with GRS for brain metastases over a 25-month period from May 2021 to May 2023 at ZAP-X Zentrum (Center) in Lingen, Germany, were examined. Inclusion criteria were age 18 years or older, brain metastasis diagnosis, history of treatment with GRS, and follow-up time of at least two months. Exclusion criteria were age under 18 years old, and a follow-up timeline of less than two months, unless the shorter follow-up time was due to death. A literature review was then performed, and comparisons between the GRS outcomes and those of other studies were carried out.

Data collected from medical records included: patient demographics, relevant previous surgical and radiation treatment, tumor characteristics, treatment plans, and follow-up data. Medical treatment prior to, during, and after GRS was also noted. Evaluation of tumor response to treatment was carried out according to the Response Evaluation Criteria in Solid Tumors (RECIST) [5] criteria, and local tumor control was assessed according to the Response Assessment in Neuro-Oncology (RANO) [6] criteria.

ZAP-X gyroscopic stereotactic radiosurgical device

The GRS device is a dedicated self-contained and self-shielded radiosurgery system developed and manufactured by ZAP Surgical Systems, Inc. of San Carlos, California [4]. The device is intended for SRS treatment of benign and malignant intracranial and cervical spine lesions (in some cases as inferior as C5). A 3.0 megavolt (MV) S-band LINAC is the source of therapeutic radiation. Like a large gyroscope, the LINAC is mounted within a combination of yoked gimbals with attached radiation shielding, each of which accurately rotates around a common isocenter. This mechanical construct enables the LINAC beam to crossfire from 2.83 π steradians of solid angle, as is ideally required for cranial SRS. Accurate therapeutic beam positioning is accomplished by these two independent isocentric rotations of the accelerator, and precise movements of a robotic patient table. Most components needed to produce the beam, such as the radiofrequency power source, waveguiding system, and beam triggering electronics, as well as significant radiation shielding, are mounted on or integrated into the rotating patient treatment chamber sphere [7,8].

The patient is supported on a movable treatment table that extends outside the treatment sphere, but which itself is also enclosed by additional radiation shielding during radiosurgery. This table shielding consists of a rotary shell and pneumatic door on a steel frame. The ZAP-X accomplishes precise three-dimensional (3D) patient registration by means of an integrated planar kilovolt (kV) imaging system that also rotates around the patient’s head. Pairs of non-coaxial X-ray images and 3D image-to-image correlation are utilized to determine the location of the patient’s anatomy with respect to the machine isocenter, both prior to and during radiosurgical treatment.

**Figure 1:**
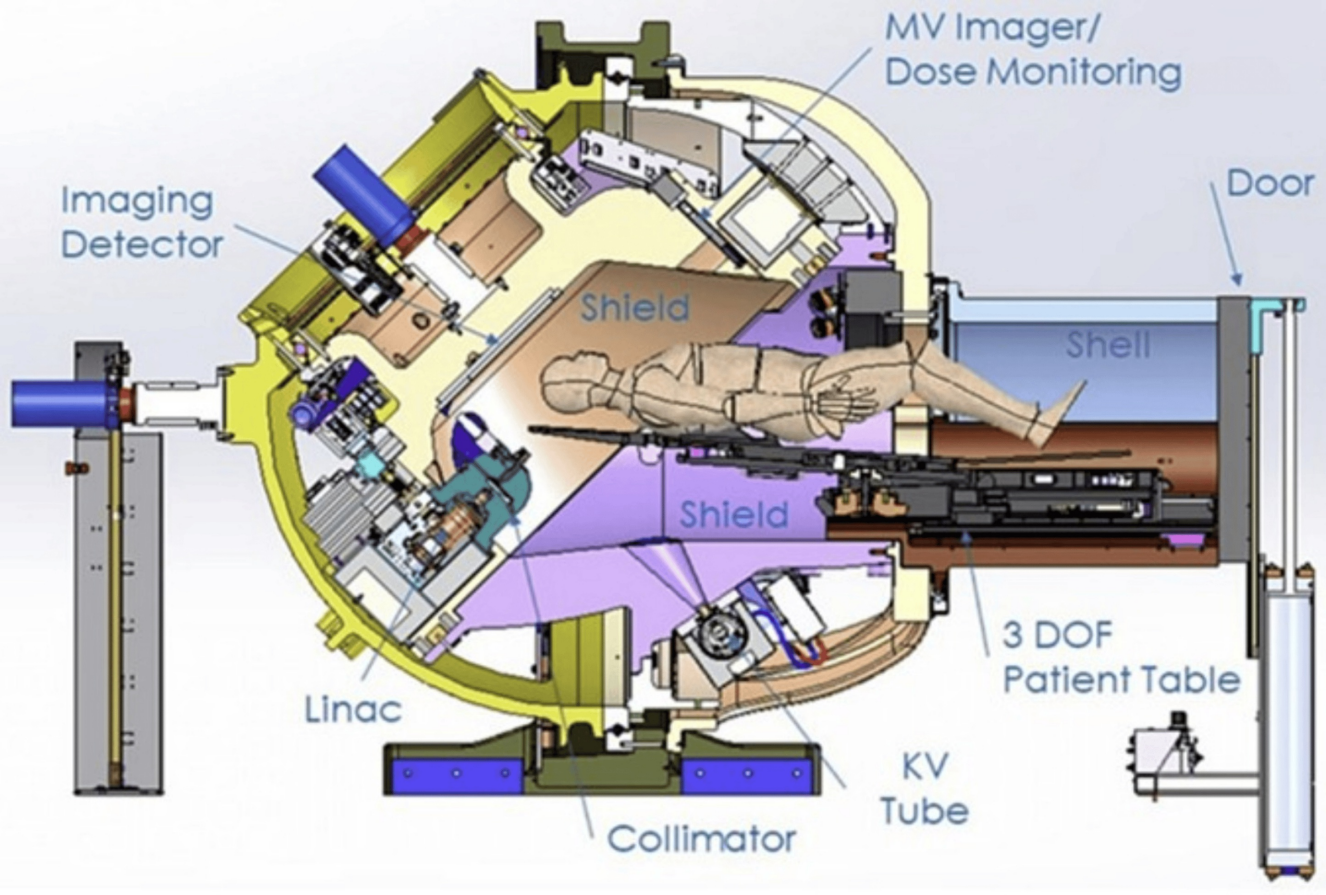
Cross-sectional view of GRS (ZAP-X) in treatment mode GRS: gyroscopic radiosurgery device; MV: megavoltage; KV: kilovotage; 3-DOF: three degrees of freedom, LINAC: linear accelerator This figure has been adapted from Weidlich et al. [8], which is an open-access article distributed under the terms and conditions of the CC-BY license.

Treatment planning is accomplished by a dedicated, purpose-designed treatment planning system that uses dosimetric sphere packing and optimizes the resulting dose distribution following a set of user-defined planning objectives governing dosimetric target coverage, dose-to-target conformance, and the sparing of critical anatomical structures. Due to the massive X-ray target shielding utilizing 5 Tenth Value Layers of Tungsten (W), the out-of-field peripheral dose fall off is rapid, and the uninvolved brain and the patient at large are extremely well shielded. A cross-section of the ZAP-X is shown in Figure 1.

Gyroscopic radiosurgery treatment details

The cohort here reported had a treatment mean target volume of 11.59 cm^3^ (0.2-36.70 cm^3^). For patients with multiple targets, the mean volume was 8.74 cm^3^ (0.4-28.80 cm^3^). Treatment dosage was done according to the center's policies. The treatment center's institutional protocol for treatment dosing is 20 Gy in naive metastatic lesions and 18 Gy in metastatic lesions previously treated with WBRT or affecting the brainstem. Seventeen patients received single fraction treatment with a dose of 18 Gy if the patient had received prior WBRT. In all other cases the prescribed dose was 20 Gy. One patient with a single metastasis of 19 cm^3^ received staged GRS with a dose of 2 x 11 Gy separated by four weeks.

The mean V10 volume dose was 10.28 Gy, and the mean V12 volume dose was 7.57 Gy. The mean Conformity Index (CI) was 2.25, and the mean Gradient Index (GI) was 3.15. All patients received periprocedural dexamethasone (3 x 4 mg PO). Patients in general tolerated the procedure well. To overcome any discomfort, midazolam 10mg IV was administered under oximetry monitoring in patients with claustrophobia (N=5 patients). Patients with bone metastasis received pain management with 7.5 mg of subcutaneous midazolam (N=3 patients). No adverse events were recorded during treatment delivery.

Statistical analysis

Demographics, tumor characteristics, clinical symptoms, and treatment modality were summarized by series and by outcomes, with continuous characteristics presented as medians with interquartile range (IQR) and categorical characteristics as counts with percentages. 

Clinical and radiological outcomes were tabulated by series, and the respective log odds of improvement were modeled as a linear function of patient characteristics in univariate logistic regression analysis. These characteristics were identified a priori and included patient age (years), sex, presence/absence of residual metastatic tumor, number of metastatic lesions, size, vascular involvement, edema, and whether other treatments were previously applied. Missing data points were censored, and pairwise deletion was implemented as appropriate. For each outcome, estimated odds ratios (OR) and 95% confidence intervals with corresponding p-values were presented as a forest plot.

The statistical analysis was performed through Kaplan-Meier analysis, and the graphical representations were generated using standard statistical software, IBM SPSS Statistics for Windows, version 29 (IBM Corp., Armonk, NY, USA).

## Results

Pre-gyroscopic radiosurgery

The charts of 46 patients (27 females and 19 males) with a total of 140 brain metastases were retrospectively reviewed. Patient and tumor characteristics are summarized in Table 1. The mean age of the patients was 63 years (range 36-82). Thirty-five patients presented with neurological symptoms. The most frequent symptoms were headache (45.7%), motor disturbances (30.4%), seizures (21.7%), visual disturbances (10.9%), sensory issues, vertigo, and speech impairment (8.7% each), and nausea/vomiting (6.5%). The average pre-procedure Karnofsky performance score (KPS) was 80. Thirty-four patients were taking corticosteroids, and nine were on antiepileptic medication before GRS treatment. The most represented primary tumors were non-small cell lung cancer (NSCLC; N=22, 47.8%), breast (N=11, 23.9%), melanoma (N=5, 10.9%), and others (N=8, 17.4%). Of tumors, 89.1% had a supratentorial location. The median target volume for treatments was 4.04 cm^3^ (0.21-19.59). Previous treatment modalities were systemic therapy (63.0%), surgery (28.3%), and radiation (47.8%). Twenty-four patients had multiple metastases with a mean of 3.3 (range 1-15) lesions. The mean sum of the long diameters in these patients is 21.8 mm (range 10-51 mm). Peritumoral edema was seen in 16 patients, and 11 patients had lesions that were causing mass effect.

**Table 1:** Patient and tumor characteristics GRS: gyroscopic stereotactic radiosurgery

Variable	Value
Number of patients (N (%))	46 (100%)
Mean age (years)	63 (range: 36-82)
Sex	
Male	19 (41.3%)
Female	27 (58.7%)
Histology	
Lung	22 (47.8%)
Breast	11 (23.9%)
Melanoma	5 (10.9%)
Others	8 (17.4%)
Tumor location	
Supratentorial	41 (89.1%)
Infratentorial	5 (10.9%)
GRS dose (Gy)	
20	28 (60.9%)
18	17 (36.9%)
2x 11	1 (2.2%)
Mean treatment time (minutes)	56.4 (range: 12.12-124.0)
Treated volume (cm^3^) - single lesion	
Mean	13.95
Range	0.2-37.30
Treated volume (cm^3^) - multiple lesions	
Mean	10.33
Range	0.4-28.80
Presenting symptoms and findings	
Headache	21 (45.7%)
Motor disturbances	14 (30.4%)
Seizures	10 (21.7%)
Sensory issues	4 (8.7%)
Nausea/vomiting	3 (6.5%)
Vertigo	4 (8.7%)
Speech impairment	4 (8.7%)
Visual disturbances	5 (10.9%)
Previous treatments	
Systemic therapy	29 (63.0%)
Surgery	13 (28.3%)
Radiation	22 (47.8%)
Pharmacotherapy	
Corticosteroids	34 (73.9%)
Antiepileptic drugs	9 (19.6%)
Conventional chemotherapy	14 (30.4%)
Immunotherapy	15 (32.6%)

Post-gyroscopic radiosurgery

The prescription dose was 18 Gy if the patient had previously received WBRT; otherwise, the dose was 20 Gy. All patients received periinterventional dexamethasone (3x4 mg PO). Radiation prescription doses ranged from 10-20 Gy, with a mean of 18.86 Gy (single fraction). The treatment time range was 12.12-124.0 minutes (mean: 56.4 minutes). Treatment V10 volume ranged from 0 to 36.7 (mean: 12.59), and treatment V12 volume ranged from 0 to 28.80 (mean: 9.47). No patients experienced any adverse events during treatment. Patient outcomes are summarized in Table 2. Mean follow-up time was 8.5 months (range 0-19). The average post-procedure KPS was 85.6, which was a general improvement in functionality. No new neurological deficits were observed, and there was no worsening of symptoms. As far as medication requirements, 36 patients continued with corticosteroids, and 10 patients continued antiepileptic drugs after GRS treatment. Nine patients required further treatment due to progressive disease. Two patients required craniotomy for resection of the tumor, and one of these patients had additional fractionated stereotactic radiation therapy. Another patient received CyberKnife (Accuray Inc., Sunnyvale, CA, USA) radiosurgery, while the other six underwent repeat GRS. Systemic response using RECIST criteria was 17.3% complete response, 10.9% partial response, 45.7% stable disease, and 26.1% progression of disease. Tumor control evaluation was performed by serial MRI imaging, with an observed control rate of 73.9% and a 26.1% progression rate of disease according to the RANO criteria for brain metastases.

**Table 2:** Patient outcomes GRS: gyroscopic stereotactic radiosurgery; RECIST: Response Evaluation Criteria in Solid Tumors; RANO: Response Assessment in Neuro-Oncology

Variable	Value
Median overall survival (months (range))	15.9 (14.1-19.0)
Progression-free survival (months (range))	9.6 (7.7-11.4)
Local tumor control (months (range))	10.7 (9.5-11.9)
Local tumor control by histology (months (range))	
Lung	12.0 (4.5-19.5)
Breast	5.0(0.0-14.3)
Melanoma	4.0 (2.9-5.1)
Others	6.0 (4.7-7.3)
Mean pre-GRS Karnofsky performance scale	80%
Mean post-GRS Karnofsky performance scale	85.6%
Adverse effects	0
Further treatment pursued (N (%))	9 (19.6%)
Cranial surgery	2 (4.3%)
CyberKnife radiosurgery	1 (2.2%)
Gyroscopic radiosurgery	6 (13.0%)
RANO criteria (N (%))	
Complete response	8 (17.3%)
Partial response	5 (10.9%)
Stable disease	21 (45.7%)
Progressive disease	12 (26.1%)
RECIST criteria (N (%))	
Complete response	8 (17.3%)
Partial response	5 (10.9%)
Stable disease	21 (45.7%)
Progressive disease	12 (26.1%)
Symptoms outcomes (N (%))	
Stable or improved	34 (73.9%)
Worsening	12 (26.1%)

**Figure 2:**
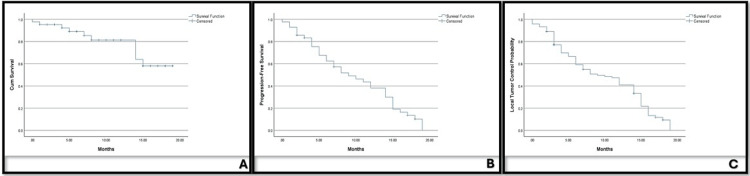
Kaplan-Meier analysis Kaplan-Meier plot of (A) overall survival, (B) progression-free survival, and (C) local tumor control. Created with IBM SPSS Statistics for Windows, version 29 (IBM Corp., Armonk, NY, USA).

Figure 2 shows the Kaplan-Meier plot for overall survival, progression-free survival, and local tumor control.

Illustrative cases

We present the following three illustrative cases of patients with different primary cancer pathologies who underwent GRS treatment for brain metastasis.

**Figure 3:**
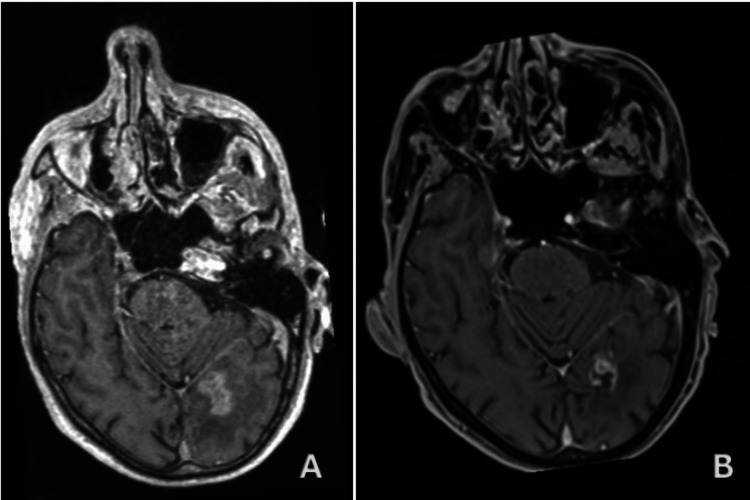
Patient A, pre- and post-GRS imaging (A) Initial imaging of recurrent metastatic lesion on pre-treatment axial T1 post-contrast MRI. (B) Complete response on post-treatment axial T1 post-contrast MRI at 17 months follow-up. GRS: gyroscopic stereotactic radiosurgery

Patient A is a 61-year-old male with a history of NSCLC, who, two years after the initial diagnosis, presented with a left temporal lesion subsequently treated with surgery and fractionated stereotactic radiotherapy (FSRT) (20 Gy), achieving a complete response according to RECIST criteria. Two years later, he presented with seizures and a KPS of 80 with imaging (Figure 3A) revealing a new left temporo-occipital 21 mm metastatic lesion. GRS was offered using device and treatment plan was set to a planning target volume (PTV) 6.3 cc, prescription dose of 18 Gy at 55.6% with 11 isocenters, delivered in one fraction. No adverse events occurred. At 18 months follow-up, the patient was seizure free with a KPS of 100 and he achieved a complete response according to RECIST criteria, and partial tumor control according to RANO criteria (Figure 3B).

**Figure 4:**
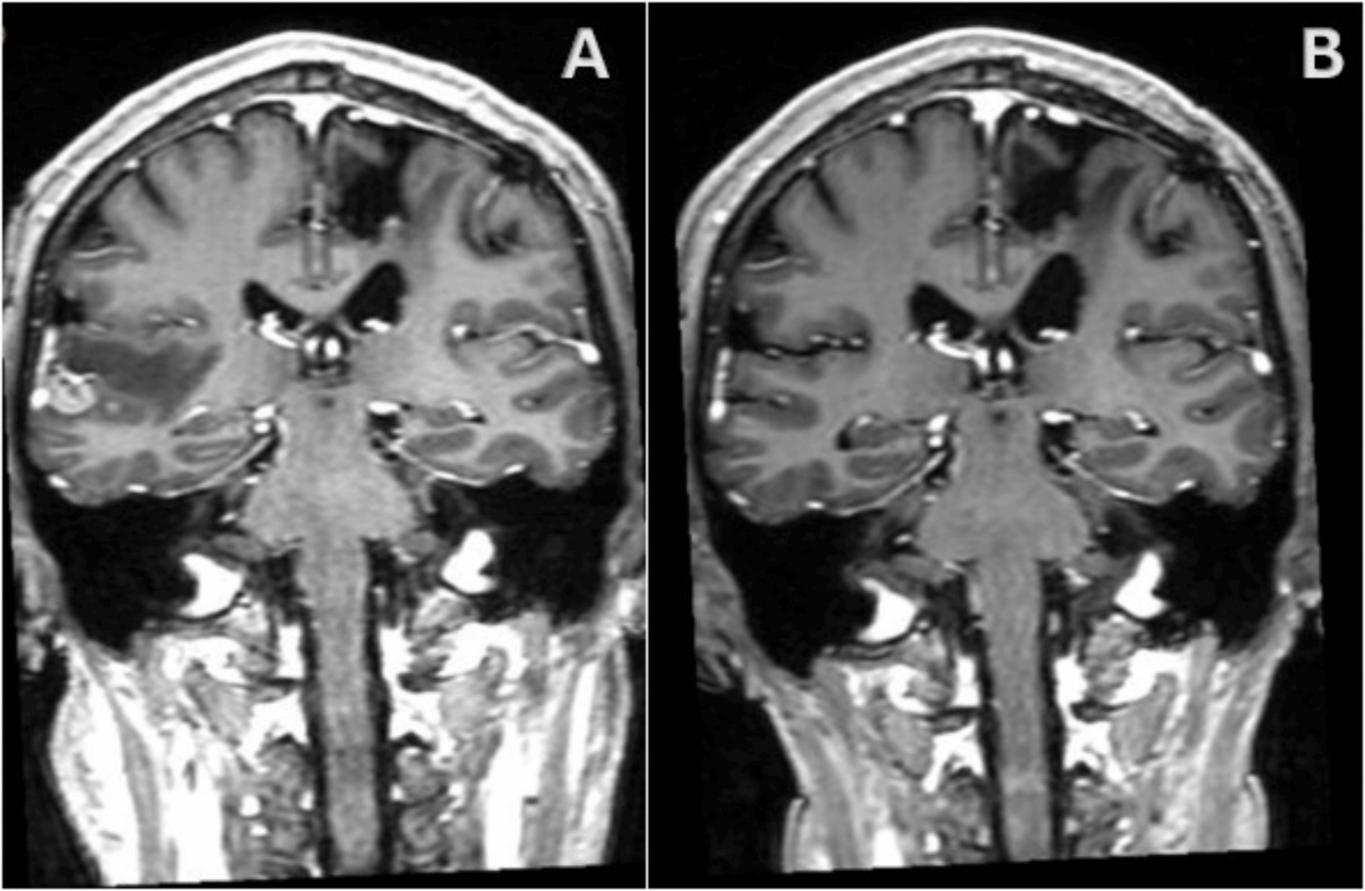
Patient B, pre- and post-GRS imaging (A) New 15 mm right temporal metastatic lesion on pre-Treatment axial T1 post-contrast MRI. (B) Complete response of the target lesion on post-treatment axial T1 post-contrast MRI. GRS: gyroscopic stereotactic radiosurgery

Patient B is a 49-year-old male with cancer of unknown primary, who underwent surgery for a left frontoparietal metastatic adenocarcinoma, achieving a complete response according to RECIST criteria. On follow-up imaging two years later (Figure 4A), a new 15 mm right temporal lesion was discovered. The patient was neurologically intact, presenting with a KPS of 100. GRS treatment was planned with a PTV volume of 2.5 cc, a prescription dose of 20 Gy at 55% isodose line with 5 Isocenters, delivered in one fraction. No adverse effects occurred during treatment. At four months follow-up imaging (Figure 4B) the patient reached a complete response to the targeted metastatic lesion, with an unchanged KPS of 100 and no neurological symptoms. However, at one-year follow-up imaging, a new right frontal lesion was identified. A second treatment with ZAP-X was scheduled to address this new brain metastasis, achieving complete response according to RECIST and RANO criteria at two months follow-up. The patient, at this point also responded well to the treatment, showing no adverse effects with a KPS score of 90.

**Figure 5:**
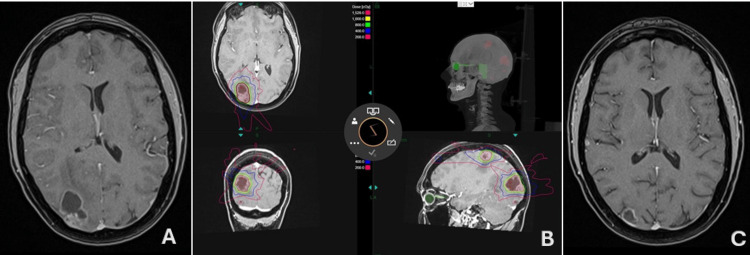
Patient C, pre- and post-GRS imaging (A) Pre-treatment axial T1 post-contrast MRI of metastatic lesions. (B) Staged GRS protocol treatment planning for two large occipital and parietal metastases. PTV of 19.3 cc treated in two fractions with a prescription dose of 20 Gy, delivered at 58.9% isodose line. (C) Post-treatment axial T1 post-contrast MRI. GRS: gyroscopic stereotactic radiosurgery

Patient C is a 44-year-old female with a history of ovarian carcinoma with two large occipital and parietal metastasis and four small temporooccipital lesions discovered six years after the primary tumor diagnosis (Figure 5A). She presented only with headache and a KPS of 70. Given the high number of target lesions, a staged GRS protocol was chosen. First, the two large lesions, accounting for a PTV of 19.3 cc, were treated with a prescription dose of 20 Gy, delivered at 58.9% isodose line in two fractions (Figure 5C). Lastly, the four small temporooccipital lesions were treated in a single session with a prescription dose of 20Gy for a PTV of 3.1 cc. No adverse effects occurred in either treatment. At seven months follow-up, all lesions exhibited a favorable treatment, reaching partial response according to RANO criteria and complete response according to RECIST: the four small lesions were not seen on MRI, whereas the two large lesions achieved a 79% volume decrease from baseline (Figure 5B). The patient displayed a positive response to GRS and no new neurological deficits were observed.

## Discussion

In the cases previously presented, the patients had an excellent response to ZAP-X treatment with complete symptomatic relief and no significant adverse effects. Although patient B had progressive disease shown by a new metastatic lesion, this was observed at a different site than the initial target, which is not uncommon for aggressive NSCLC pathologies. Patient C serves as a compelling example of the safety and efficacy of GRS in addressing multiple targets. The GRS outcomes we presented with the three illustrative cases and those observed in the whole patient cohort do not differ from those reported in patients who have undergone treatment with traditional SRS devices [9]. This demonstrates that the GRS safety and efficiency are comparable to other conventional SRS devices.

GRS is a new, image-guided LINAC-based, self-shielding radiosurgery system that does not require a vault. It is specifically designed to deliver image-guided, focused radiation with a dedicated 3 megavolt linear accelerator without the need for a radiation-safe vault [7,8,10]. 

Past reviews have found preliminary evidence of the feasibility, tolerability, and short-term safety of the ZAP-X Gyroscopic Radiosurgery System [11-13]. Authors have described the need to further examine the clinical outcomes of patients who have undergone this novel treatment [14-16]. To our knowledge, the cohort described in this study is the largest one published to date. In Table 3, we provide a comparative review of the handful of publications regarding patients with brain metastasis that have been treated with GRS that are available at the time of this publication.

**Table 3:** Comparative table of previous studies of GRS for brain metastasis GRS: gyroscopic stereotactic radiosurgery

Article number	Title of article	Authors/year of publication	Type of study	Number of patients	Number of metastatic lesions	Number of targets	Median prescription dose (Gy)	Number of fractions	Mean treatment time (minutes)	Median follow-up time (months)	Median overall survival (months)
1	Self-shielding gyroscopic radiosurgery: a prospective experience and analysis of the first 100 patients	Ehret et al. (2024) [17]	Prospective observational study and retrospective comparison	Not specified	76	1-10	20 (18-22)	1	47 (19-197)	N/A	N/A
2	Gyroscopic stereotactic radiosurgery: a retrospective analysis	Conroy et al. (2025) [18]	Retrospective case series	30	98	1-11	24 (12-35)	1-5	24 (9-123)	14.1	20.3
3	Self-shielding gyroscopic radiosurgery- a first clinical experience, case series, and dosimetric comparison	Muacevic et al. (2022) [11]	Retrospective case series	7	13	N/A	20 (19-21)	1	30 (26-34)	N/A	N/A
4	Zap-X gyroscopic radiosurgery system: a preliminary analysis of clinical applications within a retrospective case series	Hendricks et al. (2022) [14]	Retrospective case series	23	Not specified	1-5	18 (12-30)	1-5	66	10.2	Not specified

In this study, we are presenting the analysis of the first treatments provided at the ZAP-X Zentrum in Lingen, Germany. We report the experience on 46 patients suffering from brain metastasis (BM) with a minimum follow-up (FU) of two months. Results suggest that GRS can be just as safe and effective as conventional SRS.

In approximately half of the studies reported, a median of 44% (range, 6.9%-100%) of patients received whole brain radiation therapy either before or within one month of SRS [19]. This percentage of additional treatments could be significantly higher if considering repeat SRS or open surgical resection. Only 19.6% of our cohort required further treatment after GRS due to progression, showing comparability with other SRS devices [4]. Studies suggest that medium-sized to larger lesions are associated with a local tumor control rate of approximately 75% and 69% respectively, and our overall tumor control rate for this cohort is 73.9% [19]. In this study, we did not evaluate potential synergies between GRS and concurrent systemic therapies, the cumulative effect of prior radiation exposure, or how these could alter the therapeutic ratio or the dose/volume/response relationship. Given the increasing availability of systematic therapies for patients with metastatic disease, it could be worthwhile to further study the efficacy of these with concurrent GRS treatment.

Limitations

Some limitations of this study are its retrospective nature and relatively small cohort. Inclusion of additional metastatic pathologies, reports on staged therapies for larger volume lesions, and matched cohort analysis would greatly impact the evaluation of GRS. However, this study significantly contributes to the growing literature regarding GRS outcomes.

## Conclusions

GRS is a safe and effective treatment modality for patients with metastatic brain disease. To our knowledge, this study represents the largest cohort reported to date of patients with brain metastasis treated with GRS. The clinical outcomes observed demonstrate that GRS can produce similar outcomes as those reported with conventional SRS. Further studies are needed to continue to longitudinally evaluate its performance across a diverse range of pathologies and patient populations. However, this study shows that GRS is a safe and feasible treatment option for patients with brain metastasis.
